# Painful Tubercle; a Report of Two Cases, and the Result of a Careful Dissection, by Which It Is Shown Not to Be a Neuroma, or an Enlargement of the Nervous Tissue, as Has Been Generally Believed

**Published:** 1852-09

**Authors:** Frank H. Hamilton

**Affiliations:** One of the Surgeons of the Buffalo Hospital


From the Buffalo Medical Journal.

*Painful Tubercle; a Report of Two Cases, and the Result of a careful Dissection, by which it is shown not to be a Neuroma, or an Enlargement of the Nervous Tissue, as has been generally believed.* By Frank H. Hamilton, M. D., one of the Surgeons of the Buffalo Hospital.

I do not remember to have met with more than the two following cases of "painful tubercle," and as the tumor is exceedingly rare, and its pathology does not seem to have been generally understood, I shall claim for its consideration some space.

Case I.—I. L. D., aged 26; from Fayette, Seneca Co.; in good health. Nine months since, he first discovered a small hard tumor on the back of the left shoulder, immediately under the skin. It was then, and has been ever since, extremely sensitive. Four months since, it was accidentally hit, and the pain was intense. It will not even bear the chafing of his clothes without pain. It never has been inflamed. It is now about the size of a large pea; hard, irregular, of a brownish color, and movable.

I removed it by excision, cutting away the cellular texture freely about it. I could not discover any nerve or other vessel communicating with it, or through it. Its texture was firm and fibrous.

Case II.—Hannah Duchett, æt. 25 years; healthy. About five years since, she began first to feel a "deep" pain, at a single point, on the fibular side of the calf of the right leg. The pain was then, and has always been since, paroxysmal, occurring at first at intervals of a month or so. The sensation was like electricity, shooting from the point, as a focus, in several directions up and down the leg, but not extending usually farther than four or five inches. Such was the pain, so peculiarly diagnostic of the "painful tubercle."

All this preceded the existence of any tumor, or of any mark by which the spot could be designated; and it was not until a year ago that she first began to perceive a very small, hard tumor, at the point of suffering.

The tumor has now attained the size of a large pea; the skin is slightly elevated, but not discolored. Upon the centre of the summit of the tumor is a small black spot, of the size of the point of a pin, which looks like the obstructed lacuna of one of the cutaneous follicles. The tumor is hard, and slightly movable. It is exquisitely sensitive; the gentle brush producing a severe pain. The pains are still paroxysmal, but recur almost daily and often many times during a single day.

May 3, 1852. I excised the tumor with a considerable amount of cellular texture around it.

*Examination of the Product.*—The tumor lay imbedded upon the under surface of the skin, in which it had formed a cup-like depression, but to which it had no attachments except a very loose cellular. The skin over it, was reduced to less than half its usual thickness. The most careful dissection, aided by a glass, on all sides of the tumor, did not disclose the smallest filament of a nerve, or any thing except the same loose cellular texture.

It was covered with three investments, formed, as I have usually found them in non-malignant tumors occurring in cellular textures, only that the number is sometimes greater, viz., *first*, a tunic formed of condensed cellular texture, the result of the gradual encroachment of the tumor upon this texture; *second*, its immediate and proper investment, which was more dense and pearly; *third*, loose flocculi, or delicate cellular structure uniting the two.

The tumor was firm and elastic. When cut it looked to the naked eye semi-cartilaginous, or firm gelatiniform. With the eye glass small white spots were visible, but no fibres. Its color was very white.

Dupuytren, in his oral lectures, first demonstrated that such tumors were not neuromata. The symptoms and pathology are explained by him in a manner so graphic that we need, I think, never be in doubt; yet it will be seen, that very few have observed his distinctions; and it is for this reason that I have thought it proper to give these cases so much in detail, as additional confirmations of the correctness of his opinions.

